# Gibberellin in tomato: metabolism, signaling and role in drought responses

**DOI:** 10.1186/s43897-021-00019-4

**Published:** 2021-11-24

**Authors:** Hagai Shohat, Natanella Illouz Eliaz, David Weiss

**Affiliations:** 1 Institute of Plant Sciences and Genetics in Agriculture, The Hebrew University of Jerusalem, P.O. Box 12, 76100 Rehovot, Israel; 2 Plant Biology Laboratory, The Salk Institute for Biological Studies, La Jolla, CA 92037 USA

**Keywords:** Gibberellin, DELLA, Abscisic acid, Drought resistance, Tomato, Gene editing, Yield

## Abstract

The growth-promoting hormone gibberellin (GA) regulates numerous developmental processes throughout the plant life cycle. It also affects plant response to biotic and abiotic stresses. GA metabolism and signaling in tomato (*Solanum lycopersicum*) have been studied in the last three decades and major components of the pathways were characterized. These include major biosynthesis and catabolism enzymes and signaling components, such as the three GA receptors GIBBERELLIN INSENSITIVE DWARF 1 (GID1) and DELLA protein PROCERA (PRO), the central response suppressor. The role of these components in tomato plant development and response to the environment have been investigated. Cultivated tomato, similar to many other crop plants, are susceptible to water deficiency. Numerous studies on tomato response to drought have been conducted, including the possible role of GA in tomato drought resistance. Most studies showed that reduced levels or activity of GA improves drought tolerance and drought avoidance. This review aims to provide an overview on GA biosynthesis and signaling in tomato, how drought affects these pathways and how changes in GA activity affect tomato plant response to water deficiency. It also presents the potential of using the GA pathway to generate drought-tolerant tomato plants with improved performance under both irrigation and water-limited conditions.

## Introduction

Drought is a common and devastating abiotic stress which causes damage to crops worldwide (Dai, 2011; Tardieu, 2020). Water deficiency directly and indirectly suppresses major biochemical pathways, including photosynthesis and primary carbon metabolism, leading to inhibition of growth, flowering and fruit development (Zhu, 2016; Tardieu et al., 2018). Plants have adopted three major strategies to cope with drought: drought escape, drought tolerance and drought avoidance (Chaves et al., 2003). Some annual plants escape from severe drought by early flowering (Kooyers, 2015). Drought tolerance is acquired by osmotic adjustment (accumulation of osmolytes), accumulation of stress-protecting proteins and scavenging of reactive oxygen species (ROS) (Vinocur and Altman, 2005). All higher (vascular) plants exhibit ‘drought avoidance’ (drought-stress avoidance) responses during transient water-deficit episode. These include rapid stomatal closure and suppression of canopy growth to reduce transpiration (Brunner et al., 2015; Lind et al., 2015). At the same time, roots continue to grow, in search of new sources of water, a phenomenon called hydro- or xero-tropism (Feng et al., 2016; Dietrich, 2018). This leads to an increased root-to-shoot ratio and improved water balance.

Phytohormones play a central role in plant responses to drought (Verma et al., 2016; Gupta et al., 2020). During the early stages of soil dehydration, the major stress hormone abscisic acid (ABA) accumulates and induces various drought responses (Cutler et al., 2010), leading, in some plants, to drought tolerance, and in all higher plants to ‘drought avoidance’ (Kooyers, 2015). Numerous studies have shown that the growth-promoting hormones, auxin (Shani et al., 2017; Salehin et al., 2019), cytokinins (Nishiyama et al., 2011, Nishiyama et al., 2011; Farber et al., 2016), brassinosteroids (Ye et al., 2017; Planas-Riverola et al., 2019; Xie et al., 2019) and gibberellins (GAs, Colebrook et al., 2014), reduce plant resistance to water deficiency.

The growth-promoting hormone GA regulates numerous developmental processes throughout the plant life cycle, from seed germination to fruit development (Yamaguchi, 2008; Daviere and Achard, 2013). GA also negatively affects plant response to biotic and abiotic stresses (Navarro et al., 2008; Colebrook et al., 2014). GA and inhibitors of GA biosynthesis are widely used in agriculture to control germination, stem elongation, plant architecture, flowering time and fruit development (Rademacher, 2016). Accumulating evidence suggest that inhibition of GA activity, either by chemical treatments or by gene-editing, can also be used to improve plant performance under stress conditions (Eshed and Lippman, 2019). Drought opposes GA-induced processes; it inhibits seed germination, shoot growth and fruit development (Munns and Tester, 2008). Several studies have shown that osmotic stress inhibits GA accumulation (Achard et al., 2006; Nelissen et al., 2018; Shohat et al., 2021). In turn, the reduced GA levels lead to the accumulation of DELLA, the master growth inhibitor, which promotes adaptation to abiotic stresses, including drought (Colebrook et al., 2014).

Tomato (*Solanum lycopersicum*), like many other crops, is susceptible to drought (Iovieno et al., 2016; Zhou et al., 2019). In the past two decades, numerous studies on tomato response to drought have been conducted (Gur and Zamir, 2004; Gong et al., 2010), including studies assessing the role of GA in such processes (Nir et al., 2014, 2017; Omena-Garcia et al., 2019; Illouz-Eliaz et al., 2020; Shohat et al., 2021). Here, we review the current knowledge on GA biosynthesis and signaling in tomato, how drought affects these pathways and how these changes in hormone activity affect tomato plant response to water deficiency. We also present the potential in exploiting the GA pathway to generate drought-tolerant tomato plants with improved performance under irrigation and water-limited conditions.

### GA metabolism and signaling

#### GA metabolism

**Fig. 1:**
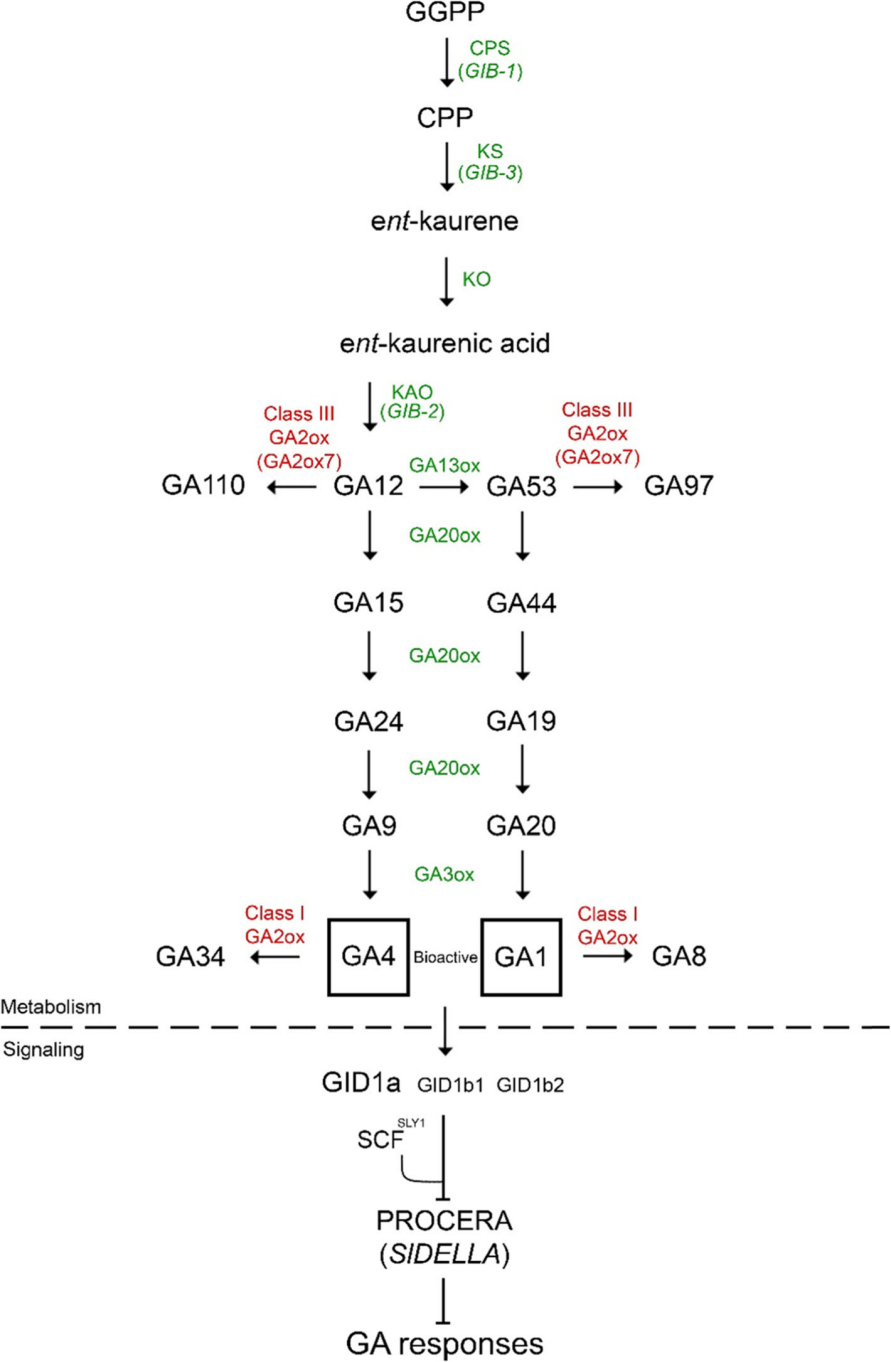
GA metabolic and signaling pathways in tomato. The scheme shows GA biosynthesis enzymes (green), GA deactivation enzymes (red) and bioactive GAs (black squares)

A comprehensive and up-to-date review on GA metabolism was recently published by Hedden (2020). GAs are diterpenoids, produced from the general substrate geranylgeranyl diphosphate (GGPP), which is converted to *ent-*kaurene by *ent-*copalyl diphosphate synthase (CPS) and *ent-*kaurene synthase (KS) in the plastids (Fig. 1). *ent-*kaurene is then converted to the first GA precursor GA_12_, by two cytochrome P450 monooxygenases, i.e., *ent-*kaurene oxidase (KO) and *ent-*kaurenoic acid oxidase (KAO), which act on the outer membrane of the plastids and in the endoplasmic reticulum, respectively. Bioactive GAs, are synthesized in the cytosol from GA_12_ and GA_53_ by two 2-oxoglutarate-dependent dioxygenases (2-ODDs) families, GA 20-oxidases (GA20ox) and GA 3-oxidases (GA3ox). GA_12_ is converted to GA_9,_ and GA_53_ to GA_20,_ by GA20oxs. Then, GA3oxs, convert GA_20_ and GA_9_ by 3β-hydroxylation to GA_1_ and to GA_4_, respectively.

GA deactivation plays a central role in the regulation of bioactive GA accumulation in response to both environmental and developmental cues (Yamaguchi et al., 2008). GA inactivation is primarily catalyzed by another family of 2-ODD enzymes, known as GA 2-oxidases (GA2ox), which reduce the levels of bioactive GAs. *GA2ox* genes are classified as either class I, which catalyze the conversion of bioactive GAs (GA_1_ and GA_4_) or their direct precursors (GA_20_ and GA_9_) to biologically inactive GA derivatives_,_ or class III, which use the early GA precursors GA_12_ and GA_53_ as substrates. Other GA deactivation mechanisms are driven by cytochrome P450s, which acts on non-13-hydroxylated GAs (GA_12_, GA_9_ and GA_4_) to produce epoxidized GAs that lack biological activity (Zhu et al., 2006), and GA METHYL TRANSFERASE1 (GAMT1) enzymes, which methylate bioactive GAs to form inactive GA methyl esters (Varbanova et al., 2007).

#### GA sensing and signaling

GA acts by triggering the destruction of DELLA (Locascio et al., 2013). While DELLAs lack a DNA-binding domain, they interact with transcription factors to activate and repress transcription (Zentella et al., 2007; Yoshida et al., 2014). GA binding to the GIBBERELLIN-INSENSITIVE DWARF1 (GID1) receptor increases receptor affinity to DELLA, leading to the formation of the GA-GID1-DELLA complex (Fig. 1). This facilitates the interaction of DELLA with an SCF E3 ubiquitin ligase complex via the GID2/SLEEPY1 (SLY1) F-box protein. The SCF^SLY1^ complex polyubiquitinates DELLA, targeting it for degradation by the 26S proteasome (Sasaki et al., 2003; Dill et al., 2004; Griffiths et al., 2006; Harberd et al., 2009; Hauvermale et al., 2012), which subsequently leads to transcriptional reprogramming and activation of GA-dependent responses.

**Fig. 2:**
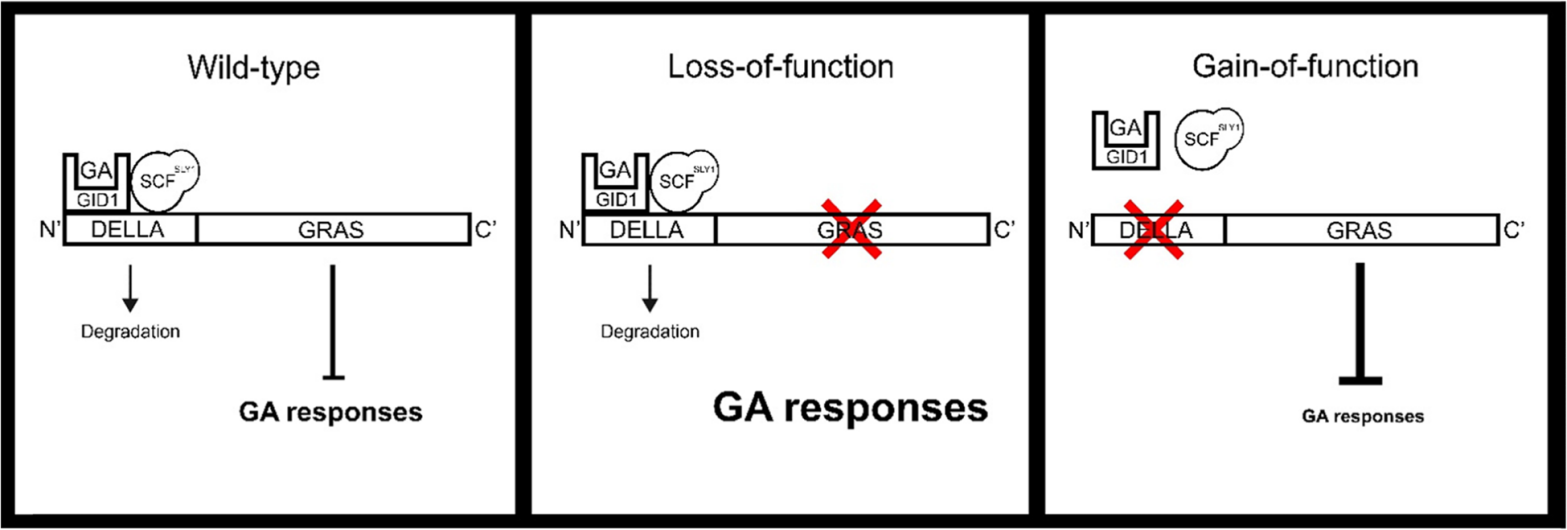
Schematic presentation of the two types of DELLA mutants and their effect on GA signaling. Wild-type (left), DELLA loss-of-function (center) and gain-of-function (right) mutations. Red X represents the mutation site in DELLA

GID1 interacts with DELLA’s N-terminal region which harbors the conserved DELLA and VHYNP motifs. The C-terminal region of DELLA interacts with various transcription factors to repress GA responses, rendering it the element responsible for DELLA activity (Sun et al., 2012; Locascio et al., 2013). Mutations in the N-terminal region of DELLA block its interaction with the GID1 receptor, thereby preventing DELLA degradation (Fig. 2). Such gain-of-function dominant mutations constitutively inhibit GA responses, including growth. Several studies have shown that these mutants are tolerant to various biotic and abiotic stresses, including drought (Magome et al., 2008; Bari et al., Bari and Jones, 2009; Nir et al., 2017). By contrast, loss-of-function, recessive mutations in the C-terminal region of DELLA are associated with constitutive GA responses (Fig. 2), resulting in excess elongation and stress-susceptible plants (Achard et al., 2006, 2008; Nir et al., 2017).

#### GA metabolism, sensing and signaling in tomato

Tomato is widely used as a model system for crop research; it is diploid, self-compatible, simple to cross, easy to grow and has an efficient transformation protocol. As a result, well characterized genetic materials and tools, sequenced genome and extensive gene expression profiles are available (The Tomato Genome Consortium, 2012). Studies in tomato cover many topics, including flowering, fruit development and maturation, secondary metabolism, interaction with the environment and hormone activity, in general, and GA metabolism and signaling, in particular (Serrani et al., 2007; Livne et al., 2015; Illouz-Eliaz et al., 2019; Israeli et al., 2019; Shinozaki et al., 2020).

The GA metabolism and signaling pathways in tomato are summarized in Fig. 1. *gib-1*, *gib-2*, and *gib-3*, three GA-deficient mutants identified and characterized in tomato (Koornneef et al., 1990; Bensen and Zeevaart, 1990) exhibit typical GA-deficiency phenotypes, including dwarfism, small and dark green leaves and delayed seed germination, all of which are corrected by application of exogenous GA (Butcher et al., 1990). *GIB-1* encodes CPS*, GIB-3* encodes KS and *GIB-2* encodes KAO (Bensen and Zeevaart, 1990; Koornneef et al., 1990). The tomato CPS, KS and KO are encoded by a single gene, and KAO, which forms GA_12_, has four paralogs (Pattison et al., 2015).

The later steps in the pathway are catalyzed by rather large families of 2-ODDs; 8 putative GA20ox, 6 putative GA3ox and 11 putative GA2ox (Pattison et al., 2015; Chen et al., 2016; Shohat et al., 2021). CRISPR-derived *ga20ox1* and *ga20ox2* mutants, recently characterized in tomato (Shohat et al., 2021), exhibit mild GA-deficiency phenotypes, including shorter stems and smaller leaves. The *ga20ox1/ga20ox2* double mutant exhibited an additive effect, including severe dwarfism, dark-green, small leaves and delayed germination, suggesting that *GA20ox1* and *GA20ox2* play a key role in GA biosynthesis in tomato. A mutation in the tomato class III GA-deactivating gene *GA2ox7* increases the levels of bioactive GA_1_ and GA_4_, and is associated with a unique phenotype, i.e., elongated internodes but normal leaves, suggesting limited stem-to-leaf transport of bioactive GAs (Schrager-Lavelle et al., 2019).

The canonical GA signal transduction pathway in tomato includes three GID1 receptors (*GID1a, GID1b1* and *GID1b2* (Illouz-Eliaz et al., 2019)), a single DELLA protein named PROCERA (PRO) and a single F-box protein, SLY1 (Jasinski et al., 2008; Illouz-Eliaz et al., 2019, 2020). *GID1a* is the dominant GA receptor with the strongest effect on stem elongation and leaf growth. In contrast, flower growth is only affected in plants bearing type B GID1 receptor mutants. The *gid1* single and double mutants exhibit almost normal growth, suggesting overlapping activities and high redundancy. Seeds of the triple *gid1* mutant (*gid1*^*TRI*^) only germinate upon embryo rescue and the plants exhibit extreme dwarfism and complete insensitivity to GA.

Three *pro* (DELLA) loss-of-function alleles were characterized in tomato (Jasinski et al., 2008; Lor et al., 2014; Livne et al., 2015). The conserved VHVID domain in the C-terminal region of PRO is required to repress GA responses (Bassel et al., 2008). A point mutation (T905 to A) in this domain, in *pro*, resulted in constitutive GA responses, leading to early germination, elongated stems and facultative parthenocarpy (Van Tuinen et al., 1999; Bassel et al., 2008). *pro*^*ΔGRAS*^, a null mutant of *PRO* (Livne et al., 2015) lacks the entire C′-terminal part of the protein, exhibits enhanced GA responses compared to *pro,* including an extremely elongated stem and obligatory parthenocarpy. Moreover, in contrast to the weak *pro* allele, *pro*^*ΔGRAS*^ is fully insensitive to paclobutrazol and GA treatments. The third DELLA loss-of-function allele was generated using Transcription Activator-Like Effector Nucleases (TALENs, Lor et al., 2014). This mutant is null and phenocopies *pro*^*ΔGRAS*^. Transgenic tomato plants overexpressing the gain-of-function stable DELLA mutant protein *proΔ17* which lacks the DELLA domain, exhibit a severe GA-deficient phenotype and GA insensitivity (Nir et al., 2017). Another gain-of-function allele was generated using CRISPR-Cas9 technology to target the DELLA domain in *pro*^*TALEN*^, turning its loss-of-function nature to gain-of-function (Zhu et al., 2019).

A CRISPR-derived tomato *sly1* mutant exhibits severe dwarfism (Illouz-Eliaz et al., 2020). *sly1* is insensitive to GA, suggesting a strong inhibition of GA signaling, confirming the importance of DELLA degradation via the proteasome pathway to relieve GA responses in tomato.

### The role of GA and DELLA in tomato plant response to water deficiency and adaptation to drought

**Fig. 3:**
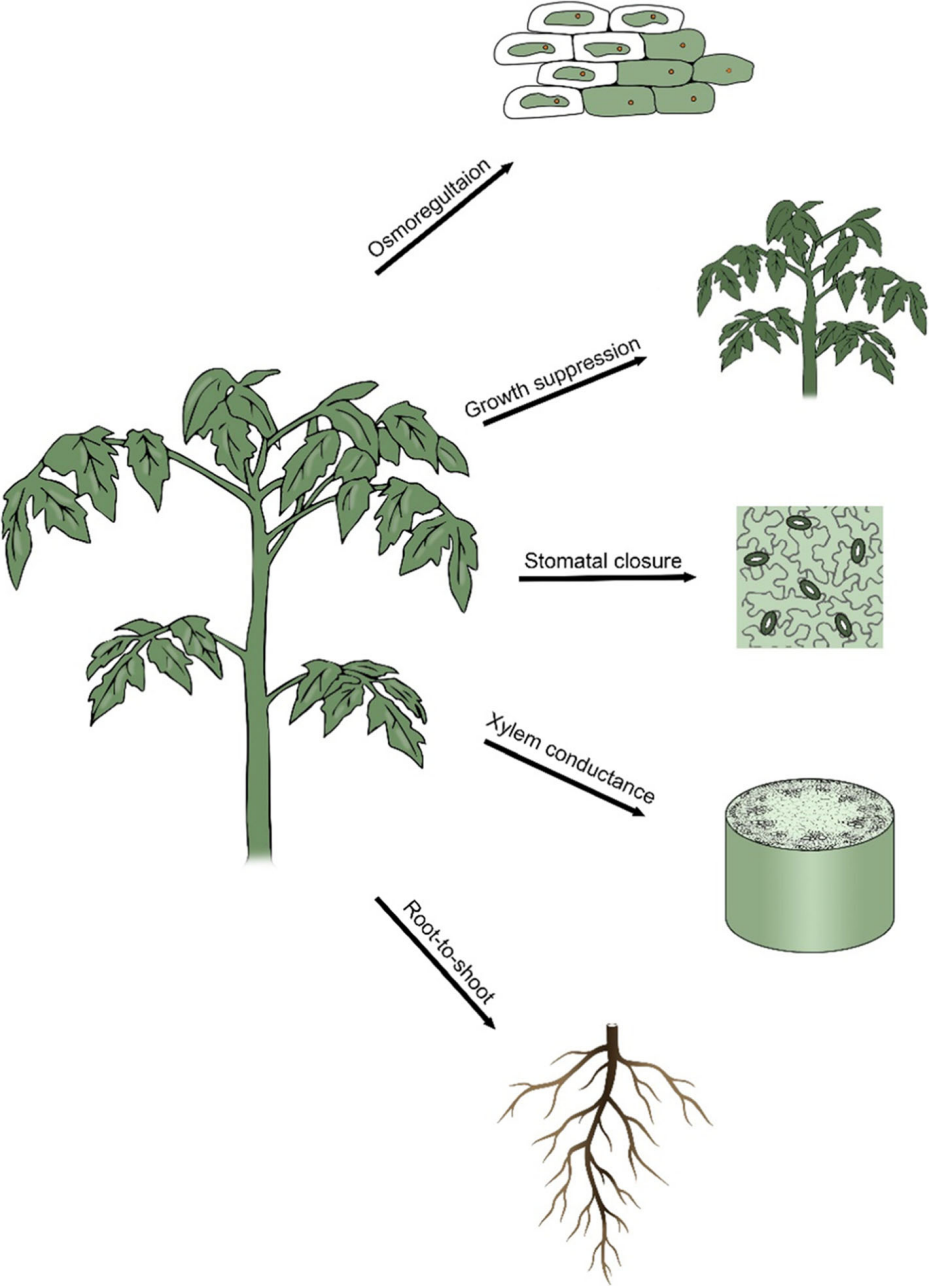
Low GA activity promotes drought resistance in tomato via several mechanisms. Low GA levels or activity promote ‘drought tolerance’ by osmoregulation (Omena Garcia et al., 2019). It also promotes ‘drought avoidance’ by inhibiting canopy growth, accelerating stomatal closure, reducing xylem expansion and proliferation and increasing root to shoot ratio (Nir et al., 2014,2017; Illouz Eliaz et al., 2020; Ramon et al., 2020; Shohat et al., 2020, 2021)

The role of DELLA in plant responses to abiotic stresses originated independently of GA; the liverwort *Marchantia polymorpha* DELLA ancestor regulates responses to stress despite the lack of GA and the canonical GA signaling pathway (Hernandez-Garcia et al., 2021). In higher plants, DELLA accumulation depends on GA and both, antagonistically, affect plant response to stress. Several studies in tomato have shown that inhibition of GA activity and accumulation of DELLA promote drought resistance by affecting several different metabolic and developmental processes throughout the plant life cycle, from seeds to mature plants (Fig. 3, Nir et al., 2014, 2017; Omena-Garcia et al., 2019; Illouz-Eliaz et al., 2019, 2020; Shohat et al., 2021).

#### GA and drought tolerance in tomato

Tomato seeds are tolerant to desiccation and can germinate after years of dry storage (Priestley et al., 1985). ABA has a central role in the acquisition of desiccation tolerance (Ooms et al., 1993; Finkelstein et al., 2008) through its promotion of the activity of various major regulators of seed desiccation tolerance during seed maturation, including *ABA INSENSITIVE3 (ABI3)*, *FUSCA3* (*FUS3*) and *LEAFY COTYLEDON1* (*LEC1*) and *LEC2* (To et al., To A et al., 2006). GA opposes ABA activity in seeds (Groot et al., 1987; Tyler et al., 2004; Steinbrecher and Leubner-Metzger, 2017), and also affects desiccation tolerance; tomato DELLA null mutant *pro*^*∆GRAS*^ seeds are susceptible to desiccation and fail to germinate even after short periods (days) of storage (Livne et al., 2015). This was attributed to the low expression of the ABA-regulated, drought tolerance-related genes *ABI3*, *FUS3* and *LE25* in *pro*^*∆GRAS*^ seeds. It was therefore suggested that the accumulation of DELLA during seed maturation is important for the acquisition of ABA-induced long-term drought tolerance in tomato seeds.

Tolerance to drought can be acquired by osmotic adjustment, i.e., the accumulation of ions and organic solutes in the cells (Shabala and Shabala, 2011). Under water-deficit conditions, some plants accumulate high levels of solutes in their roots and leaves to reduce the cellular osmotic potential and maintain high turgor pressure (Turner, 2018). Omena-Garcia et al. (2019) reported that the GA-deficient *gib-1, gib-2 and gib-3* tomato mutants accumulate higher levels of osmolytes, and were able to maintain higher leaf water content and leaf turgor under water-deficit conditions.

#### GA and ‘drought avoidance’ in tomato

All higher plants respond to water limitation by rapid stomatal closure and inhibition of shoot growth (Brunner et al., 2015). These responses reduce transpiration and water loss (Skirycz and Inzé, 2010). Nir et al. (2014) showed that inhibition of bioactive GA accumulation in tomato by overexpressing the Arabidopsis *GAMT1* gene, reduces water loss under drought conditions. The reduced transpiration in the transgenic plants was ascribed to the smaller leaves and to reduced stomatal aperture. Later, Nir et al. (2017) showed that overexpression of the stable DELLA protein *pro∆17* in tomato plants reduced stomatal aperture and transpiration, independently of leaf growth. Moreover, targeted overexpression of *pro∆17* in guard cells was sufficient to reduce stomatal aperture, suggesting that PRO acts in guard cells in a cell-autonomous manner. In line with this, the DELLA loss-of-function *pro* mutant exhibits increased stomatal conductance and water loss under water-deficit conditions. This effect of GA/DELLA is likely part of the natural ‘drought avoidance’ response in tomato; under water-deficit conditions the expression of the GA deactivation gene *GA2ox7* is strongly upregulated in guard cells, leading to reduced levels of bioactive stomatal GAs (Shohat et al., 2021). This upregulation of *GA2ox7* is required for the rapid stomatal response to drought, as the loss of GA2ox7 activity inhibited stomatal closure in the early stages of soil dehydration (Shohat et al., 2021). A role for GA in stomatal movement was also described in *Commelina benghalensis, Vicia faba* and *Fritillaria imperialis*, where GA application increased stomatal aperture (Santakumari and Fletcher, 1987; Goring et al., 1990).

The effects of *pro∆17* on stomatal closure and water loss were suppressed in the ABA-deficient *sitiens* (*sit*) tomato mutant, indicating that the effect of DELLA is ABA-dependent. While DELLA did not affect ABA levels, increased DELLA activity promoted ABA responses in guard cells (Nir et al., 2017; Shohat et al., 2020). RNAseq analysis of isolated guard cells derived from tomato plants with high versus low DELLA (PRO) activity, identified the ABA transporter *ABA-IMPORTING TRANSPORTER 1.1* (*AIT1.1*) as upregulated by PRO (Shohat et al., 2020). The CRISPR-derived *ait1.1* mutant exhibits increased transpiration and reduced ABA-induced stomatal closure. *ait1.1* also suppresses the promoting effect of DELLA on stomatal closure, suggesting that most, if not all, of the effects of GA/DELLA on stomatal response to water deficiency are related to the negative cross-talk between GA and ABA.

GA and DELLA also impact ‘drought avoidance’ through developmental responses. Reduced transpiration throughout prolonged periods of water deficiency is also achieved by growth suppression and the reduction of transpiration area (Salah and Tardieu, 1997). Several studies suggest that inhibition of GA accumulation under water-deficit conditions plays a role in drought-induced growth suppression (Skirycz and Inzé, 2010; Litvin et al., 2016). For example, low levels of GA in *Populus* inhibit growth and promote resistance to water-deficit conditions (Zawaski and Busov, 2014). Drought conditions inhibit GA accumulation in maize leaf elongation-zones and suppress their growth (Nelissen et al., 2018). The reduced GA levels in tomato under water-deficit conditions is a results of both, inhibition of GA biosynthesis and activation of GA catabolism (Litvin et al., 2016; Shohat et al., 2021). Water-deficit conditions inhibit the expression of the GA biosynthesis genes *GA20ox1* and *GA20ox2*, promote the expression of *GA2ox7*, reduce the levels of bioactive GAs and suppress leaf expansion (Shohat et al., 2021). *ga20ox1* and *ga20ox2* mutants exhibit reduced whole-plant transpiration under water-deficit conditions due to their smaller canopy area.

While shoot growth is inhibited under water-deficit conditions, root growth is maintained, and even promoted, leading to increased root-to-shoot ratio (Sharp et al., 2004). These developmental changes improve water balance under water-limited conditions. Some evidence implies that GA has a role in altering root-to-shoot ratio under water-deficit conditions. Although GA promotes root elongation in Arabidopsis (Yaxley et al., 2001; Fu and Harberd, 2003), in some other species, GA has no effect or even suppresses root growth (Berova and Zlatev, 2000; Gou et al., 2010; Fonouni-Farde et al., 2019; Moriconi et al., 2019). Reduced GA levels or signaling promote lateral root density and growth in *Populus* (Gou et al., 2010). In *Medicago*, GA inhibits and the GA biosynthesis inhibitor paclobutrazol, promotes primary root elongation and lateral root counts (Fonouni-Farde et al., 2019). The DELLA loss-of-function *sln1* barley mutant exhibits reduced root growth (Moriconi et al., 2019). In tomato, GA has a strong effect on shoot growth, but only a minor effect on primary root elongation (Ramon et al., 2020). In line with this, *gid*^*TRI*^ exhibits a dramatically increased root-to-shoot ratio due to the strong inhibition of shoot growth, but only a mild effect on root elongation. Thus, inhibition of GA accumulation upon water deficiency is expected to restrict shoot growth without conferring an effect on root elongation and therefore, may contribute to the increased root-to-shoot ratio.

Low GA activity also reduces water loss in tomato through changes in the hydraulic conductivity; low GA activity in *gid1a* or *sly1* mutants inhibits xylem-vessel expansion and proliferation and reduces hydraulic conductivity (Illouz-Eliaz et al., 2020). Under severe drought conditions, the effect of low GA activity on xylem expansion can also protect plants from cavitation and embolism (Ishihara and Hirasawa, 1978; Baum et al., 1999; Brodribb and Hill, 2000). Thus, inhibition of xylem expansion and proliferation by low GA activity may be another mechanism through which reduced GA promotes adaptation to prolonged periods of limited water.

### Harnessing the GA pathway to improve tomato performance under water-limited conditions

Manipulation of the GA pathway has enormous potential in crop improvement (Eshed and Lippman, 2019). Mutations in the GA biosynthesis or signaling pathways have been used to improve crops. The best example is the introduction of semi-dwarf cereal crops in the 1960s, which led to a significant increase in yield. The semi-dwarf varieties are resistant to lodging even when excessively fertilized (Wu et al., 2020). Two major types of mutations are responsible for what has come to be known as the ‘Green Revolution’ (Hedden, 2003); a loss-of-function mutation in the *SD1* gene encoding the GA biosynthesis enzyme *GA20ox2* in rice (Monna et al., 2002; Sasaki et al., 2002; Spielmeyer et al., 2002), and a gain-of-function mutation in *Rht1*, a gene encoding DELLA in wheat (Peng et al., 1999).

As described above, the GA pathway can also be harnessed in tomato to enhance resistance to abiotic stresses, including drought. Since GA and DELLA have a pleotropic effect on growth, a trade-off between yield and drought resistance is expected. However, this might only be true for strong inhibition of GA activity. Illouz-Eliaz et al. (2020) showed that while mutation in a single GA receptor (GID1a) suppressed growth in the field, it had no effect on yield, giving rise to a tomato line with a higher harvest index (fruit weight/plant fresh weight). This is a desired side-effect of GA inhibition, in that it allows higher planting density to obtain higher yield per unit area (Gifford and Evans, 1981). Thus, the ultimate goal is to generate mutants with mild dwarfism, normal yield under well-watered conditions and significantly improved drought resistance. Introduction of the CRISPR technology has made this more feasible to achieve within a relatively short time (Jinek et al., 2012; Brooks et al., 2014), in contrast to the decades required when using classical breeding (Bai and Lindhout, 2007). CRISPR-based technologies provide a variety of genome-editing tools, including targeted mutation knockouts (KOs), tissue-specific KOs, multiplex gene editing, targeted insertion, gene activation and precise genome editing (Brooks et al., 2014; Rodríguez-Leal et al., 2017; Zhu et al., 2020; Dong and Ronald, 2021; Pan et al., 2021). CRISPR has already been applied to improve the agronomical traits of an orphan *Solanaceae* crop (*Physalis pruinosa*) and a wild tomato species (*Solanum pimpinellifolium*), by simultaneously editing four genes involved in plant architecture (SP), flowering time (SP5G) and fruit size (SlCLV1/3 and SlWUS) (Lemmon et al., 2018; Li et al., 2018).

#### Possible GA pathway targets for CRISPR-based mutagenesis to increase drought resistance in tomato

**Fig. 4:**
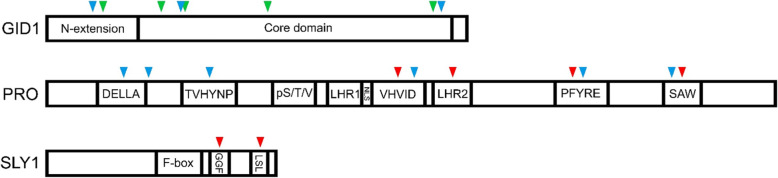
Possible target sites in the major tomato signaling components to attenuate without eliminate GA responses. GA-GID1 interaction sites are indicated by green arrows, GID1-PRO interaction by blue arrows and PRO-SLY1 interaction by red arrows. The specific sites in homologous GID1, DELLA and SLY1 are elaborated in McGinnis et al., (2003), Murase et al., (2008) and Hirano et al., (2010)

Transduction of the GA signal is based on a cascade of interactions, i.e., GA with GID1, GID1 with DELLA and DELLA with SLY1. The possible interaction sites between these three signaling components are presented in Fig. 4, and described elaborately by McGinnis et al. (2003), Murase et al. (2008) Hirano et al., (2010). Attenuating without eliminating the affinity between these interacting components, may lead to mild growth suppression without affecting yield, but with increased drought resistance. A rapid and efficient way to do so is by applying precise CRISPR-based genome-editing tools such as base editing (single base-pair substitution/deletion, Zhu et al., 2020).

Perturbations of GA binding to GID1 can be obtained by site-specific mutations in the GA binding “pocket” of GID1 (Murase et al., 2008). Attenuating the affinity of GID1 to DELLA (PRO) can be achieved by mutations in the GID1 N-terminal extension (N-Ex) domain (Murase et al., 2008) or by mutations in the N-terminal region of PRO (GID1 binding site). However, deletion of PRO’s N-terminal causes severe dwarfism (Zhu et al., 2019). Thus, mutations in other sites, outside the N-terminal region, that affect GID1 binding, may generate weak gain-of-function alleles, as shown before in rice (Hirano et al., 2010). A mild reduction in GA signaling can also be obtained by interfering with the DELLA-SLY1 interaction. SLY1 has two conserved domains required for its interaction with DELLA, i.e., the GGF domain and the LSL domain (McGinnis et al., 2003). A CRISPR-derived tomato *sly1* mutant, which carries a single nucleotide insertion, causing a frame shift and premature stop codon before the LSL domain, was already generated, but has a severe dwarf phenotype (Illouz-Eliaz et al., 2020). Using the same precise editing techniques to generate weak *sly1* alleles which only reduces the affinity to DELLA, may provide fine-tuning of GA responses only.

Attenuation of GA signaling can also be achieved using multiple guide constructs to target various cis-regulatory elements in the promoters of *GID1s, PRO* or *SLY1*. This is expected to generate a collection of alleles exhibiting changes in the expression levels and patterns and their subsequent activity, and to enable selection of drought-tolerant lines. It should be noted, however, that DELLAs are primarily regulated at the post-translational level (Blanco-Tourinan et al., 2020). Thus, this approach seems to be more relevant to GID1s and SLY1.

The GA signaling components in tomato are encoded by a small number of genes (single *PRO* and *SLY1* and three *GID1*s). Thus, mutation in a single gene might lead to undesired phenotypic changes and yield loss. Illouz-Eliaz et al. (2019, 2020) show that although the *gid1a* mutant grows well under stable conditions, it exhibits phenotypic instability when grown under extreme, unstable environmental conditions in the field, leading, in some plants, to strong growth suppression and yield loss. Growth and yield instability might be prevented if the target gene belongs to a large family. For example, the enzymes in the later stages of the GA biosynthetic pathway are encoded by rather large gene families in all plant species. The tomato genome encodes 8 *GA20ox*s and 6 *GA3ox*s (Pattison et al., 2015). Several studies show that enzymes from these groups exhibit tissue-specific expression (Serrani et al., 2007; Chen et al., 2016). According to their spatial expression pattern (http://bar.utoronto.ca/efp_tomato/cgi-bin/efpWeb.cgi), *GA20ox1* and *GA20ox2* seems to be the best candidates for the generation of drought-resistant plants with no, or a weak effect on yield. Indeed, *ga20ox1* and *ga20ox2* mutants exhibit a mild growth phenotype and reduced water loss under drought conditions (Shohat et al., 2021).

In conclusion, manipulations of the GA pathway in tomato can be exploited to improve drought resistance, as well as resistance to other abiotic and biotic stresses. Alongside resistance, these modifications may improve yield through their effect on plant architecture and harvest index. Further research will still be necessary to develop high-yield tomato plants with improved stress resistance using the GA pathway, and will be made possible using the recent advances in gene-editing technologies.

## Data Availability

All data presented in this review is included in this published manuscript.

## Abbreviations

2-ODDs: 2-oxoglutarate-dependent dioxygenases
ABA: Abscisic acid
ABI3: ABA INSENSITIVE3
AIT1.1: ABA-IMPORTING TRANSPORTER 1.1
CLV: CLAVATA
CPS: Copalyl diphosphate synthase
CRISPR: Clustered regularly interspaced short palindromic repeats
FUS3: FUSCA3
GA: Gibberellin
GA13ox: GA 13-oxidase
GA2ox: GA 2-oxidase
GA20ox: GA 20-oxidase
GA3ox: GA 3-oxidase
GAMT1: GA METHYL TRANSFERASE1
GGPP: Geranylgeranyl diphosphate
GID1: GIBBERELLIN INSENSITIVE DWARF 1
KAO: Kaurenoic acid oxidase
KO: Kaurene oxidase
KOs: Knockouts
KS: Kaurene synthase
LEC: LEAFY COTYLEDON
N-Ex: N-terminal extension
PRO: PROCERA
ROS: Reactive oxygen species
SD1: SEMIDWARF 1
*sit*: *sitiens*
SLY1: SLEEPY1
SP: SELF-PRUNING
SP5G: SELF-PRUNING 5G
TALEN: Transcription activator-like effector nucleases
WUS: WUSCHEL
